# Online webinar training to analyse complex atrial fibrillation maps: A randomized trial

**DOI:** 10.1371/journal.pone.0217988

**Published:** 2019-07-03

**Authors:** João Mesquita, Natasha Maniar, Tina Baykaner, Albert J. Rogers, Mark Swerdlow, Mahmood I. Alhusseini, Fatemah Shenasa, Catarina Brizido, Daniel Matos, Pedro Freitas, Ana Rita Santos, Gustavo Rodrigues, Claudia Silva, Miguel Rodrigo, Yan Dong, Paul Clopton, António M. Ferreira, Sanjiv M. Narayan

**Affiliations:** 1 Department of Cardiology, Centro Hospitalar de Vila Nova de Gaia/Espinho, Gaia, Portugal; 2 Stanford Cardiovascular Institute, Stanford University School of Medicine, Stanford, California, United States of America; 3 Department of Cardiovascular Medicine, Stanford University, Stanford, California, United States of America; 4 Department of Cardiology, Centro Hospitalar de Lisboa Ocidental–Hospital de Santa Cruz, Carnaxide, Portugal; 5 Department of Internal Medicine, Centro Hospitalar de Lisboa Ocidental–Hospital de São Francisco Xavier, Lisboa, Portugal; 6 Universitat Politècnica de València, Valencia, Spain; University of Auckland, NEW ZEALAND

## Abstract

**Background:**

Specific tools have been recently developed to map atrial fibrillation (AF) and help guide ablation. However, when used in clinical practice, panoramic AF maps generated from multipolar intracardiac electrograms have yielded conflicting results between centers, likely due to their complexity and steep learning curve, thus limiting the proper assessment of its clinical impact.

**Objectives:**

The main purpose of this trial was to assess the impact of online training on the identification of AF driver sites where ablation terminated persistent AF, through a standardized training program. Extending this concept to mobile health was defined as a secondary objective.

**Methods:**

An online database of panoramic AF movies was generated from a multicenter registry of patients in whom targeted ablation terminated non-paroxysmal AF, using a freely available method (Kuklik *et al*–method A) and a commercial one (RhythmView–method B). Cardiology Fellows naive to AF mapping were enrolled and randomized to training vs no training (control). All participants evaluated an initial set of movies to identify sites of AF termination. Participants randomized to training evaluated a second set of movies in which they received feedback on their answers. Both groups re-evaluated the initial set to assess the impact of training. This concept was then migrated to a smartphone application (App).

**Results:**

12 individuals (median age of 30 years (IQR 28–32), 6 females) read 480 AF maps. Baseline identification of AF termination sites by ablation was poor (40%±12% vs 42%±11%, P = 0.78), but similar for both mapping methods (P = 0.68). Training improved accuracy for both methods A (P = 0.001) and B (p = 0.012); whereas controls showed no change in accuracy (P = NS). The Smartphone App accessed AF maps from multiple systems on the cloud to recreate this training environment.

**Conclusion:**

Digital online training improved interpretation of panoramic AF maps in previously inexperienced clinicians. Combining online clinical data, smartphone apps and other digital resources provides a powerful, scalable approach for training in novel techniques in electrophysiology.

## Introduction

Portable diagnostic devices and mobile health (mHealth) are powerful technologies in healthcare[1,2]. Together, they have altered the paradigm in which students and healthcare providers train and acquire new competencies[1,3–5],[6], with a generally positive impact in education and practice[6,7], and are likely to become a dominant mode of clinical training. Nevertheless, few mHealth apps or e-learning platforms have been applied to clinical electrophysiology (EP), with existing tools mostly relating to heart monitoring systems[8], ECGs and medical calculators[9,10].

We applied the paradigm of digital online training and visualization to the novel and challenging task of interpreting complex panoramic AF maps, such as those obtained from multipolar intracardiac electrograms. Briefly, electrical signals detected by intracardiac electrodes, placed in either one or both atria, yield electrograms that are then used to generate electroanatomic atrial shells (a digital reconstruction of the atrium electrical activity), which can be analyzed as maps.

The clinical relevance of panoramic AF mapping is evidenced by the plethora of methods already available, or being introduced, including focal impulse and rotor mapping (Abbott)[11,12], body surface mapping (CardioInsight, Medtronic; EP solutions Inc)[13,14], Cartofinder[15,16], Kardium[17], Acutus Medical[18] and CardioNXT[19]. Recent meta-analyses show the promise of targeting ablation to localized source regions identified by such maps, although some results were mixed[20,21]. Complex AF maps frequently have suboptimal inter-observer agreement[22], which may contribute to negative studies[23], yet few tools exist to improve map interpretation. Tools designed to compare maps from multiple approaches/technologies, on data from the same patient, are also lacking. It remains unclear if these factors contribute to variable results between centers.[20,23,24]

We set out to (1) perform a randomized trial of standardized online webinar training for this complex EP task, to read AF maps and identify potential sources where ablation terminated non-paroxysmal AF (primary objective); and (2) extend this paradigm by developing a Smartphone App to train on AF movies created by different mapping techniques (a validated free online AF method and a commercial method) on the same panoramic EP data (secondary objective).

## Methods

### Clinical AF cases

From a multicenter registry (COMPARE-AF, NCT02997254) we created an online database of downloadable movies generated by (i) a freely available AF mapping tool (Kuklik *et al*) [22,25] and (ii) a commercial method (RhythmView, Abbott Electrophysiology, CA, USA). Although used for the same purpose, these techniques differ in several key aspects that have been discussed elsewhere[22]. For the purpose of simplification, Kuklik method and RhythmView will be referred to as Methods A and B, respectively.

For this study, we identified 16 cases, in each of which ablation at an identified region terminated non-paroxysmal AF prior to PV isolation.

**Fig 1:**
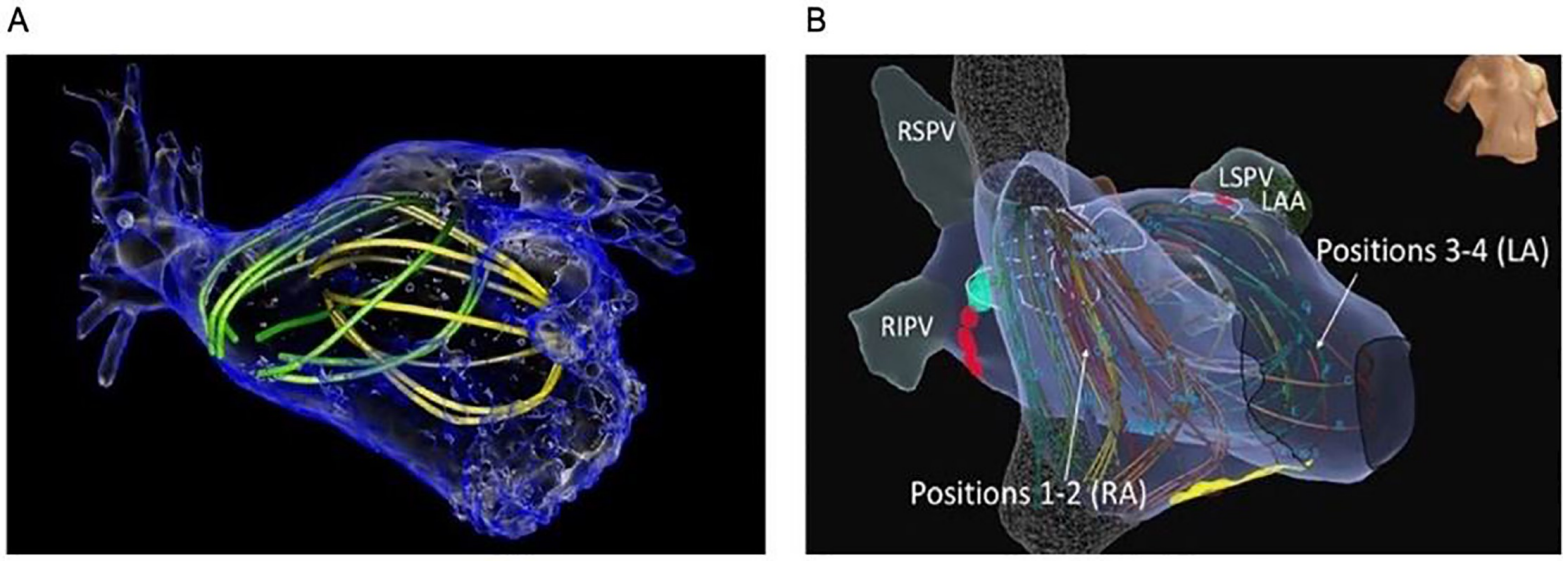
Integration between the electrical signals recorded from multiple basket positions and (A) computorized tomography of the left atrium or (B) anatomical maps.

Every patient had panoramic mapping using a basket catheter (Abbott, CA, USA)[26], moved to multiple atrial positions in AF, in successive periods. Electroanatomical shells were then created, thus correlating basket electrodes locations to atrial anatomic regions. This process allows for AF electrical signals to be recorded for the majority of both atria, after which they can be integrated with computorized tomography and/or anatomic shells information (Fig 1)[22,27].

Ablation at sites of focal or rotational activity was then performed and the site where ablation terminated AF was recorded on the electroanatomic shell, prior to pulmonary vein isolation (PVI), and referenced to electrodes on the multipolar basket.

Unipolar electrograms were exported from electrographic recorders at each site (Prucka, GE, LabSystemPro, Boston Scientific; Siemens) for analysis with the freely available Method A (http://narayanlab.stanford.edu –the code and data are freely available at the Stanford website, with no payment required. Registration is required per University guidelines)–this approach avoids proprietary methods and has been previously described[22]. Briefly, algorithms first reduce noise in unipolar signals, then remove QRS complexes and use rate-based analyses to identify activation times. Phase is then used to identify potential focal and rotational sources, with results similar qualitatively and quantitatively to results from commercial mapping systems[22,25]. Method B was a commercial reference (RhythmView) used to generate clinical maps that led to AF termination in these clinical cases. Online maps were fully de-identified and conformed to HIPAA regulations.

Participants provided an informed verbal consent for entering this trial. This project was conducted in accordance with *Stanford University Human Research Protection Program* Policy Manual, which serves as the primary source document for the IRB policies and procedures.

### Panoramic AF mapping database

In the COMPARE-AF study, all maps corresponded to patients in which source (or driver)-guided ablation terminated AF. Accordingly, whenever ablation at a pre-specified site terminated AF, this was noted and recorded as an accurate AF source. This was a linear process for maps showing only 1 source but did not fully apply whenever several sites were targeted and ablated, i.e. a patient could have had 3 sites marked for ablation, with AF terminating only after targeting the 2^nd^ or 3^rd^ site. Therefore, in order to establish the *gold standard* regarding sources location, maps were annotated for the database by 6 experts, who evaluated each movie to identify potential AF sources (blinded to clinical records and site of termination by ablation). Annotation was based on identifying rotational or focal activity if present for >3 cycles in a spatial region bounded by 2 electrodes[28]. To minimize the impact of interobserver variability, only AF sources that were identified by at least 4 out of 6 experts were regarded as valid and included. Furthermore, although some of the COMPARE-AF registry maps had a single source site identified, if at least 4 out of 6 experts agreed on specific additional sites, these would therefore be regarded as *true/present*.

Maps were further classified as either *straightforward* (>50% concordance) or *difficult* (<50% concordance), which were then split uniformly to construct separate training and testing AF movie sets.

### Assessing the impact of webinar training: Randomized trial and enrollment

**Fig 2:**
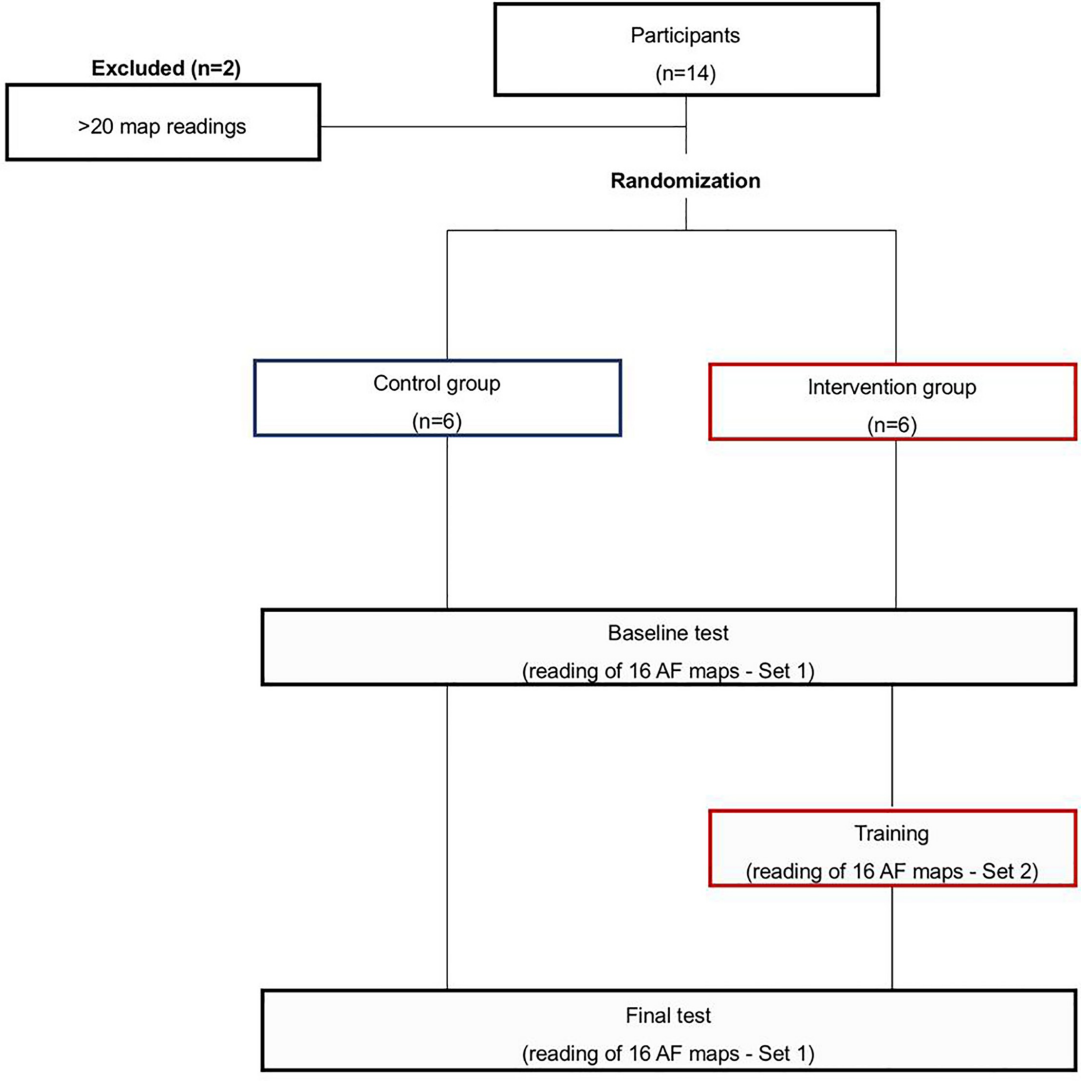
Trial design.

The impact of training was assessed by a randomized controlled trial enrolling participants who were fellows-in-training in clinical cardiac electrophysiology programs in 3 centers in Europe (n = 9), United States (n = 4) and South America (n = 1). The only exclusion criterion was meaningful previous exposure to interpreting AF maps (>20 case reads) which excluded 2 participants (Fig 2). The remaining 12 participants were included. Stanford Arrhythmia Clinic (Stanford Cardiovascular Medicine, CA, US) coordinated this trial.

**Fig 3:**
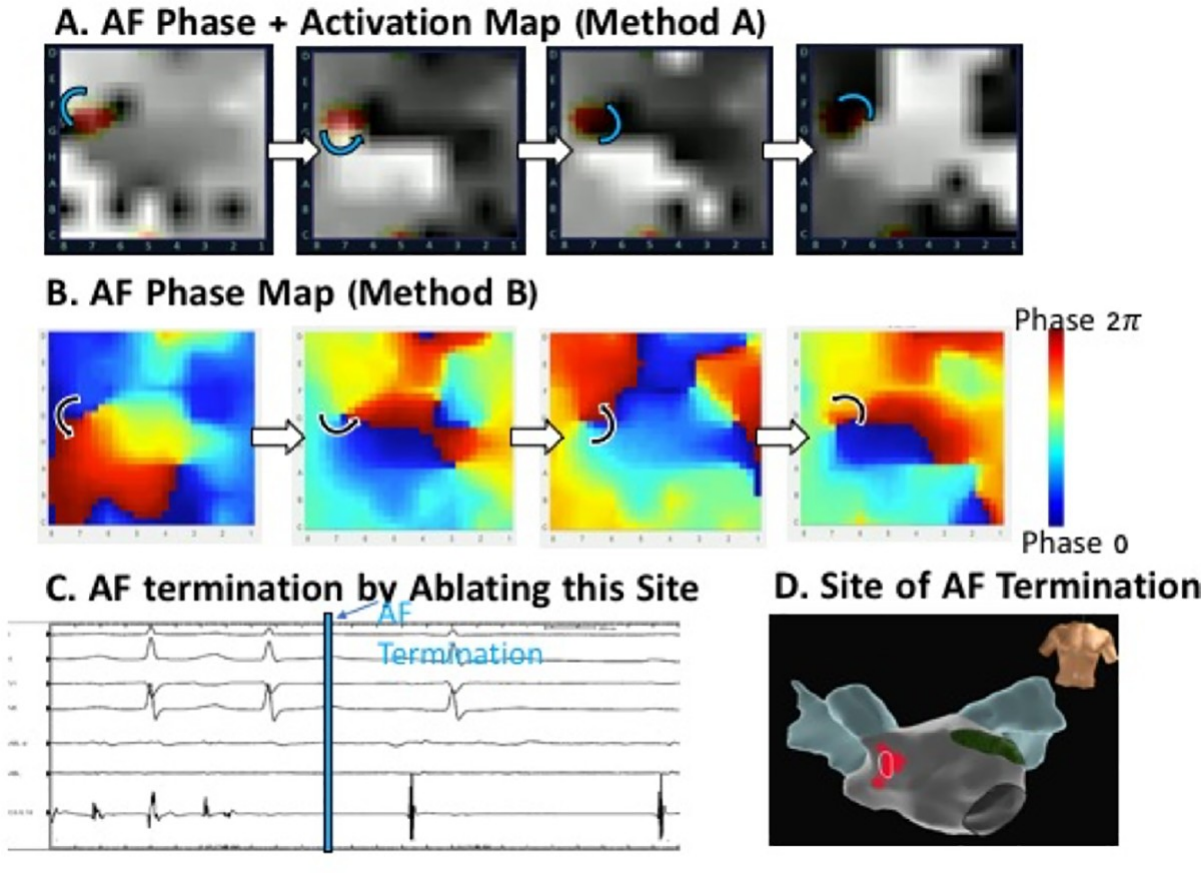
AF maps by methods A and B at site where ablation terminated persistent AF. **A.** Successive maps of AF by clinical method A shows counterclockwise rotational site (marked) which was identified prospectively during the case and targeted for ablation, but which may be difficult for an inexperienced reader to interpret. **B.** Method B analysis of same electrograms, using open-access method B, showing counterclockwise rotation. **C.** Abrupt termination of persistent AF by prospective ablation at this site prior to pulmonary vein isolation. **D.** Anatomical location of this site anterior to right pulmonary veins.

Participants were randomized to training (intervention) or no training (control) (Fig 2). All participants received a brief introduction to localized AF sources, namely types of sources (e.g. focal, rotational and rotational pattern), and how to localize them on AF movies in terms of grid coordinates. Participants took a baseline test consisting of 16 panoramic AF movies (set 1) in which they were required to identify potential rotational or focal sources validated by an EP study in the registry (Fig 3). They were blinded to correct answers, except with the knowledge that each map had at least 1 source, up to the reported maximum of 3 in each atrium (S1 Video). Subsequently, the intervention group was given a fully automated training session, in which fellows were asked to grade an entirely different set of 16 movies (set 2), shown the correct rotor or focal source location(s) and then reviewed maps. Participants then took a final test, consisting of the initial baseline set of 16 maps shuffled into a new sequence.

To prevent participants from obtaining any kind of external training or acquire information that could potentially have an impact on their final test results, all sessions took place in a consecutive fashion, i.e. participants in the control group scored the initial test, took a 1 hour break and then scored the final test. The same timeline applied to the training group, with the addition of the training set in-between the initial and final tests.

### Tests and intervention

Test (set 1) and training (set 2) webinar sessions were built using Microsoft PowerPoint (Microsoft, Redmond, WA, USA) and presented online. Both sets of maps comprised 16 movies– 8 of each mapping method (A and B, respectively)–for the same patients. Map complexity was pre-established, and 4 *straightforward* and 4 *difficult* maps were selected for both methods, with each movie playing 4 times. Participants were given an online answer sheet to submit immediately after each test. Participants randomized to training also viewed an additional set of movies (set 2): twice before the correct answer was provided, then they were shown the correct answers, after which they re-reviewed movies twice more. Participants had no control over any parameter (speed, zoom, number of replays) in both testing and training. During training, exposure to maps progressed from easier to more difficult.

**Fig 4:**
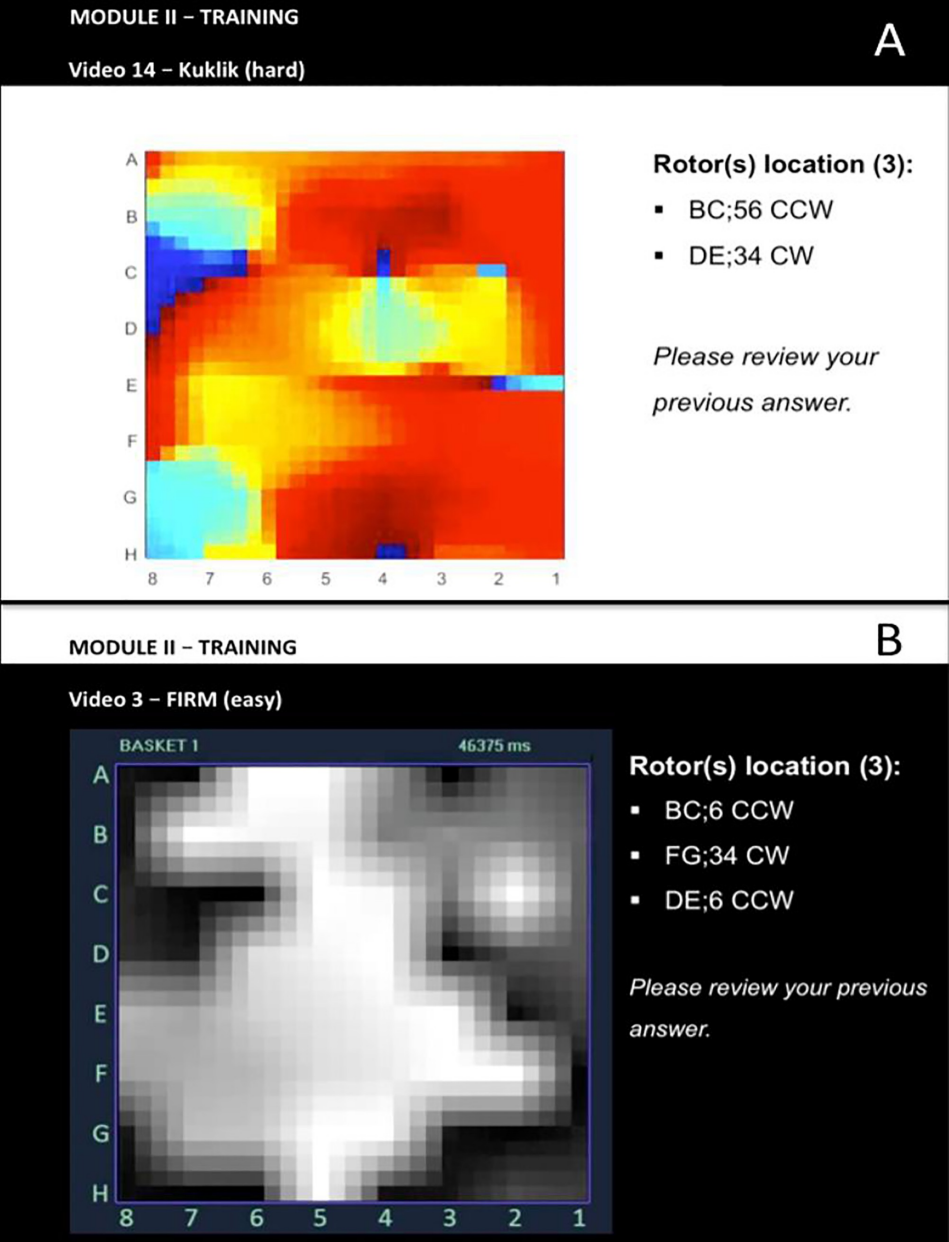
Examples of webinar training, depicting snapshots of mapping method, map difficulty, correct (expert-defined) source location and review request. (A) Method A (Kuklik *et al)*; (B) Method B (RhythmView). Participants were first presented a panoramic AF map movie (S1 Video) and asked to identify rotor location using grid coordinates and to assess rotation/turning direction; After the 3^rd^ replay, participants were shown the correct answers and asked to review their responses.

All maps were presented as grids (Figs 2 and 3; S1 Video), and potential sources were assigned locations as X_1_;Y_1_ to X_2_;Y_2_. Since rotational sources may show precession (spatial meander) for 1–2 electrodes in each direction of the grid[28], the response provided by each participant was considered correct if it was within 1 electrode coordinate of the correct location (Fig 4; S1 Video). Test score (%) was based on the total number of correctly identified sites divided by the total number of sites. The testing set had a total of 37 potential AF sources (18 in maps by method A and 19 in maps by method B)– 86% rotors and 14% focal impulses.

### Randomized trial endpoint

Comparison between initial and final test scores within each group and between control vs intervention was used to assess the impact of training versus no training. A secondary analysis of these same scores for each subset of maps (both methods) was performed to compare baseline and learning curves between methods.

### Development of online smartphone training platform

We migrated digital training to a mobile platform, developing code in Swift for iPhone7 (Xcode 8.3) to (i) access the same de-identified AF movies placed on a secure online server, (ii) display maps for each case between different mapping systems, and (iii) enable users to identify potential sources and then score them as in the Webinar. The mHealth App thus parallels each function of training performed in the clinical trial.

### Statistical analysis

Normally and non-normally distributed variables were expressed as mean±standard deviation and median (interquartile range (IQR)), respectively. Paired and independent samples T-test were used to assess differences either within or between groups, respectively. The ANOVA test and Pearson’s correlation with a two-tailed test of significance were used to evaluate differences between average test scores by different methods and between groups. Video game exposure was evaluated as a dichotomous (<1 hour/week vs ≥1 hours/week) and continuous variable (average playtime/week). Statistical analysis was performed using Statistical Package for Social Sciences (SPSS) version 24.0 (SPSS Inc., Chicago, IL) for Mac OS. Statistical significance was set at a P-value <0.05 (two-sided).

## Results

### Generation of panoramic AF maps

Panoramic AF maps were generated from 16 cases, randomly selected from a multicenter registry of 103 patients (COMPARE-AF registry), in whom ablation terminated persistent AF (NCT 02997254). This subpopulation size was selected to provide sufficient cases for training and testing without the risk of fatigue by participants. Table 1 provides clinical data for the population used in this registry.

**Table 1:** Clinical characteristics of patients contributing to the database.

Characteristic	Entire cohort	Testing set of maps (8 patients)	Training set of maps (8 patients)	P-value
Age–years (IQR)	64 (55–70)	67 (56–77)	62 (52–66)	NS
Male sex—no. (%)	82 (80)	6 (75)	7 (88)	NS
Prior AF ablation	55 (52)	4 (50)	3 (38)	NS
Left atrial diameter (mm)	42 (35–48)	47 (45–53)	48 (45–52)	NS
Left ventricle ejection fraction (%)	55 (50–60)	55 (38–60)	57 (51–65)	NS
CHADS-VASc score	2 (1–3)	2 (1–3)	3 (2–4)	NS
Termination by ablating AF source				
To sinus rhythm—no. (%)	62 (60)	2 (25)	2 (25)	
To atrial tachycardia—no. (%)	31 (30)	4 (50)	5 (63)	NS
To atrial flutter—no. (%)	10 (10)	2 (25)	1 (12)	

Each case had an average of 1.7±0.7 and 2.0±0.7 rotational sources using both mapping methods.

All experts accurately identified the termination site in >80% of the maps selected for the testing set (results obtained from their original COMPARE-AF registry maps scores). On the first (testing) set of maps, experts scored an average 78%±8% for method A and 80%±8% for method B, respectively, significantly outdoing participants’ scores in the test (p<0.001 for both methods).

### Clinical trial

Table 2 summarizes the baseline characteristics of the participants enrolled in this trial [median age 30 years old (IQR 28–32), 50% females]. Groups were well-balanced for aspects that may affect online training, such as prior video game use. Twelve fellows-in-training were enrolled and performed a total of 480 map readings: 384 for testing and 96 for training.

**Table 2:** Trial participant characteristics.

Characteristic	All patients (n = 12)	Randomization		P-value
		Control (n = 6)	Training (n = 6)	
Age–years (IQR)	30 (28–32)	31 (30–33)	28 (26–30)	NS
Male sex—no. (%)	6 (50)	3 (50)	3 (50)	
Previous exposure to activation/voltage maps—no. (%)	7 (58)	4 (67)	3 (50)	
Previous exposure to online medical training evaluation—no. (%)	10 (83)	5 (83)	5 (83)	
At least 1 gaming hour/week—no. (%)	5 (42)	2 (33)	3 (50)	
Weekly gaming time—hours (mean±SD) ^(*)^	2.1±2.2	1.3±0.4	2.7±2.9	
European based Fellows-in-training—no. (%)	9 (75)	3 (50)	6 (100)	

(*) calculated for participants that play at least 1 hour per week.

### Baseline accuracy for identifying AF source sites

**Fig 5:**
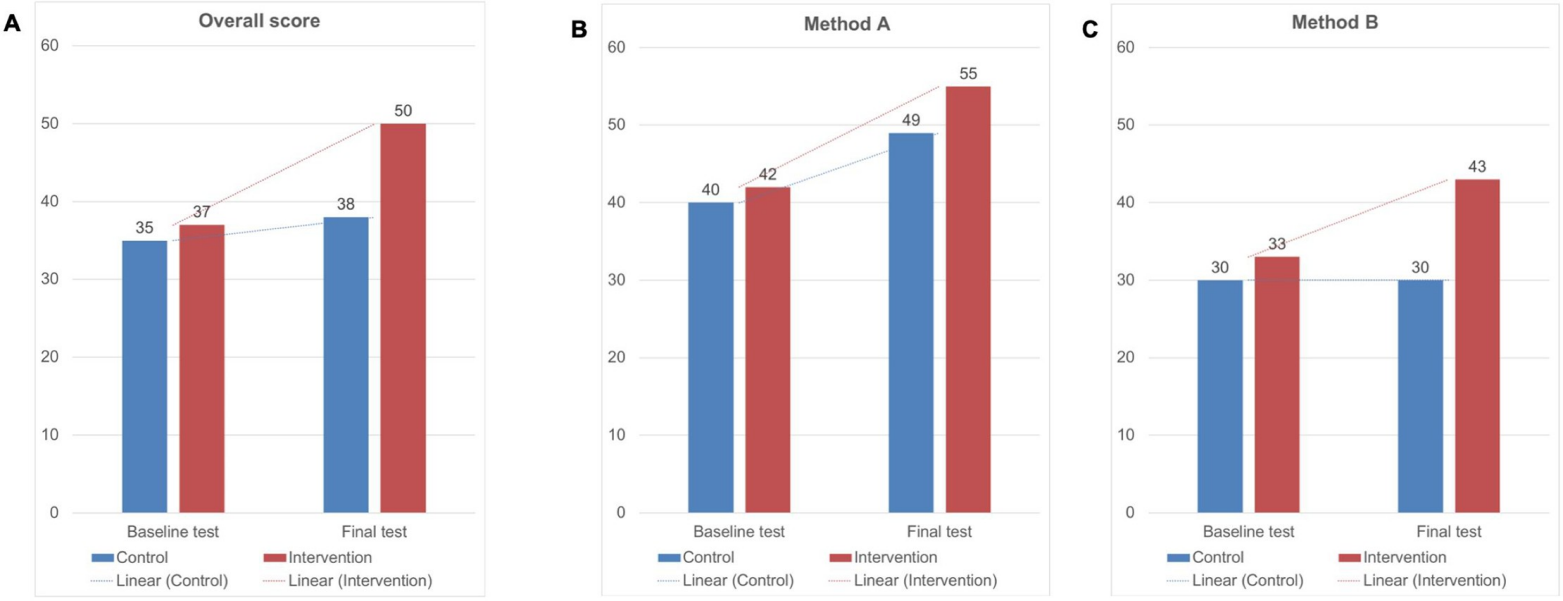
Graphical representation of (A) overall, (B) method A (non-proprietary), (C) method B (clinical) baseline and final test results for the intervention and control groups.

Overall, baseline identification of AF source sites by trial participants was relatively poor, as shown in Fig 5, but similar for control and training groups [35%±8% vs 37%±10%, respectively, (P = 0.741)]. This was true for both mapping methods–either non-proprietary method A (40%±12% vs 42%±11%, P = 0.780) or commercially available method B (30%±10% vs 32%±11%, P = 0.675). Both groups scored higher at baseline for the non-proprietary than the commercial method (P = 0.038 for training; P = 0.143 for control).

### Final accuracy for identifying AF source sites

Final test score for the training group was 50.0%±8.2% vs 37.8%±10.5% for the control group (P = 0.040). This was true for method A (54.6%±10.8% vs 49.1%±11.9%; P = 0.285) and method B (43.0%±6.2% vs 29.8%±8.6%, P = 0.021) as shown in Fig 5. Thus, the impact of training was to improve overall performance (13.1%±3.6%, P<0.001) while accuracy did not change for first- and second-reads in the control group (2.7%±3.4%, P = 0.439).

For subsets, diagnostic accuracy for method A (non-proprietary system) rose by 13.0%±4.5% (P = 0.001 vs baseline) in the training group vs 9.3%±6.7% in controls (P = 0.110 vs baseline). For method B (commercial system), training increased scores by 10.5%±6.7% (P = 0.012) vs 0%±4.7% (P = 0.600) in controls. The mean delta comparison for method B subset score was superior for the intervention group (P = 0.010), while improvement on method A was comparable between both groups (P = 0.290).

### Parallel operation of smartphone app for panoramic AF map visualization

**Fig 6:**
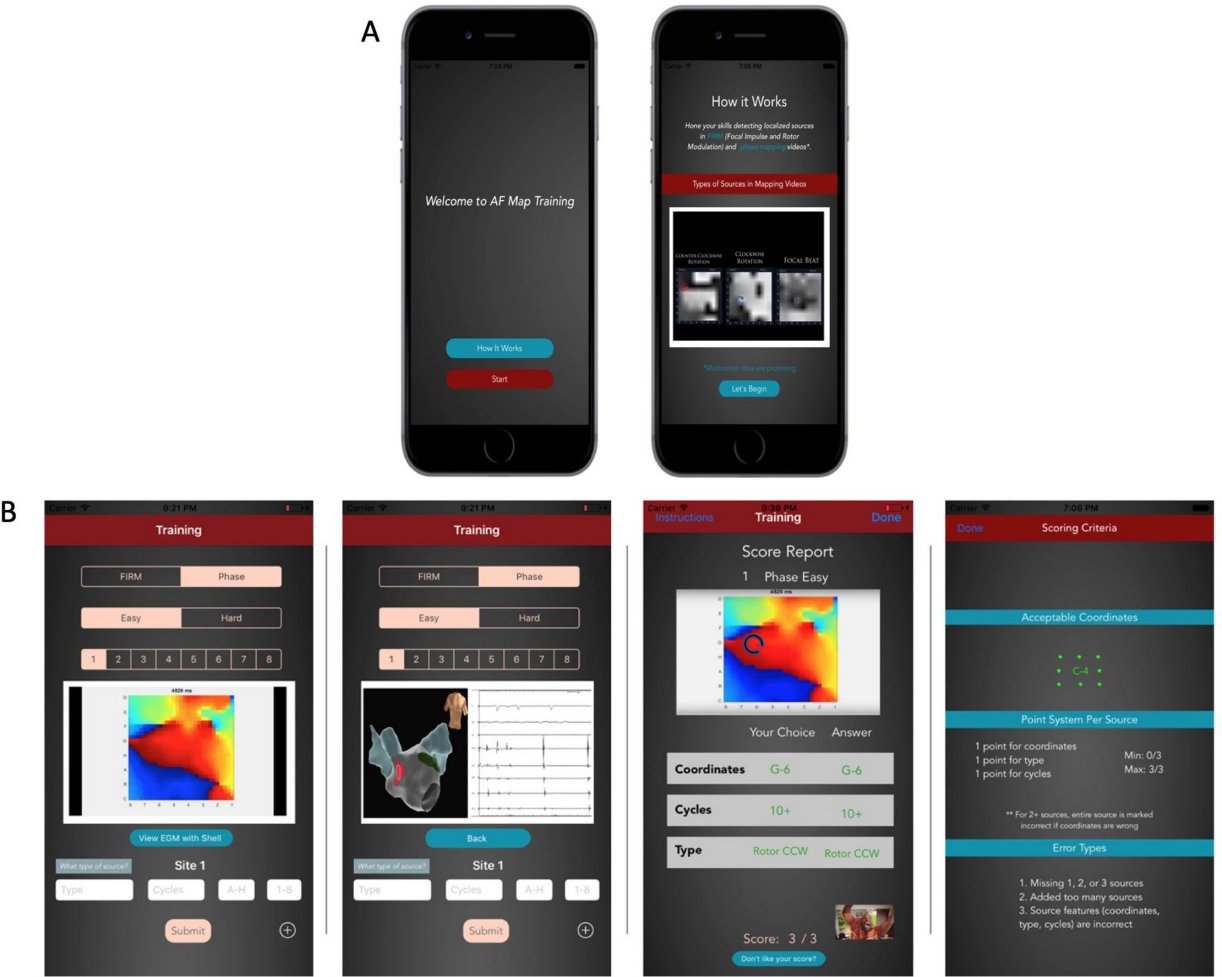
Custom smartphone map for cloud-based training. Software application (App) accesses AF movies from de-identified cloud database, displays them for user visualization, and scores their performance for online training (S1 Video). A. Introduction to smartphone app: Guidance video on commercial AF maps. B. Smartphone App Displaying Same Case as Fig 3 for Testing. In sequence, panels show video which can be played multiple times (S1 Video), with clinical data (shell, electrograms). Scoring fields are provided to grade the participant as correct or incorrect and the scoring guidelines are outlined below.

Fig 6 shows operation of the iPhone app. Fig 6A shows the introductory notes and guidance video when the App is first launched. Fig 6B shows the App displaying the case depicted in Fig 3, where ablation terminated persistent AF. This includes clinical information such as stored anatomic shells and electrograms. Once the user views the map, he/she can input their predicted site information based on their knowledge and previous training. The App grades their submission and displays the video again for review. This process continues for as long as the user wishes to train.

### Correlation with improved performance

Test results and learning curves were unaffected by gender, nationality, year-in-training and training center. However, participants who played >1 hour of video games per week showed higher diagnostic accuracy that non-gamers in every test, for either mapping method, both within each group as well as in the entire cohort (S1 Table). Although not powered to assess these differences, higher current exposure to video games was associated with improved global performance in the control group (p = 0.041), mainly driven by method A results (p = 0.010). Final test scores were always higher for gamers in the intervention group, either globally (p = 0.003) or for each method–A (p = 0.067) and B (0.033). No other factors were associated with improved diagnostic accuracy on each mapping method.

## Discussion

The main findings in our study were that: (1) graded systematic exposure to an online automated webinar improves identification of AF sources on panoramic AF maps. Nevertheless, performance on this complex task remained suboptimal, suggesting that a longer training curve is needed for optimal clinical performance; (2) Videogame exposure, but no other characteristic, was associated with higher map reading accuracy; (3) Migration of this training paradigm to a Smartphone App is feasible. Our project thus demonstrates the feasibility of several novel digital strategies to improve complex but clinically relevant visual tasks in electrophysiology. This includes the application of downloadable mapping software to compute complex AF movies from de-identified clinical electrograms, to visualize these maps to automate training, and to migrate this digital strategy to a smartphone for mobile health applications. With further development, these and similar strategies could become part of standardized training platforms in EP.

### Updating the electrophysiology training paradigm

Several aspects strengthen the study protocol and training applied in this study. First, (1) maps were validated cases of AF termination by targeted ablation at identified source sites, representing data that may not be universally available for training; (2) each movie that was placed on the secure server for the digital webinar had been reviewed by experts from a multicenter AF registry, and (3) the digital webinar was readily migrated to a custom Smartphone App. This is being made freely-available for download (http://narayanlab.stanford.edu) and could be improved for extended functionality.

A review of PubMed and MEDLINE databases from January/2000 to June/2018 yielded no results for the keywords of EP *training app* or *e-learning* software tested in a randomized controlled fashion. After extending our search to a broader range of medical specialties–namely all of those listed in the PubMed/MEDLINE database–only one anesthesiology smartphone App trial was identified[29] although results were incomplete. Although not randomized, a recent controlled study by Baumgart *et al* showed that tablet computer based integrated training and clinical practice enhanced medical education and exam performance[30]. This paucity of interactive training tools for clinical training starkly contrasts with the heavy smartphone use reported by medical students and physicians[10,31]. This may be major limitation of current medical training which, if addressed, may increase efficiency and potentially efficacy of training. In Cardiology, App distribution is markedly disproportionate, most relating to heart monitors and ECG interval calculators[9] and representing only a small percentage of total smartphone usage[10].

Furthermore, since most apps targeting health providers are unregulated, their reliability needs to be ascertained and proven true. This will prove challenging in the near future, where medical training App development will likely need to undergo the same procedures for verification, validation and certification such as any library-based resource[32].

### Digital online training as a prototype for complex EP tasks

Our aim was to develop a prototype for digital training that physicians could easily access to improve their diagnostic skills. To test this hypothesis we selected the topic of AF ablation guided by sources. While AF source ablation may improve freedom from AF when combined with PVI at several centers[11,33], testing at some centers yields conflicting results[34–36]. Interestingly, studies reporting disappointing outcomes were relatively small in size or were performed in centers new to each mapping technique. It is plausible that the learning curve for AF map reading played a role in this variability of reported outcomes[37]. Closing this training gap is hindered by the absence of certified tools.

Enrollment therefore targeted individuals who were motivated to learn and may potentially use such tools, yet none of whom had had significant prior experience with this technique (panoramic AF mapping), to avoid interfering with learning curve effects. These inclusion/exclusion criteria rendered Cardiology/EP Fellows-in-training the target population of our study. When developing the training module, we applied 2 prerequisites: (1) intervention had to be remotely delivered (e-learning) and standardized for all trainees and (2) had to consist of a graded systematic exposure to both mapping methods.

We were able to collect data from a large number of map readings and termination sites, which reduced the sample size and thus the burden of training for participants. Results showed that training improved map reading accuracy overall and for each mapping method, compared to control participants who did not receive webinar training. The experience handicap was made clearer when comparing participants’ scores to those obtained by experts, even after training. This suggests a steep learning curve for panoramic AF mapping, and further supports that interobserver variability may contribute to the inconsistent results from the efficacy of ablation targeting strategies.

### Videogame impact on the accurate interpretation of complex AF maps

A growing body of evidence is strengthening the association between time spent playing videogames and (i) enhanced visuospatial attention throughout the visual field[38] and (ii) increased perceptual reaction times, without decreased performance accuracy[39].

The visual complex characteristics of rotational sources paired with data that visuospatial working memory reduces in older adults[40] may explain why videogames correlated so strongly with test scores, even on a small group of individuals.

### Smartphone paradigm to access and display online AF maps

**Fig 7:**
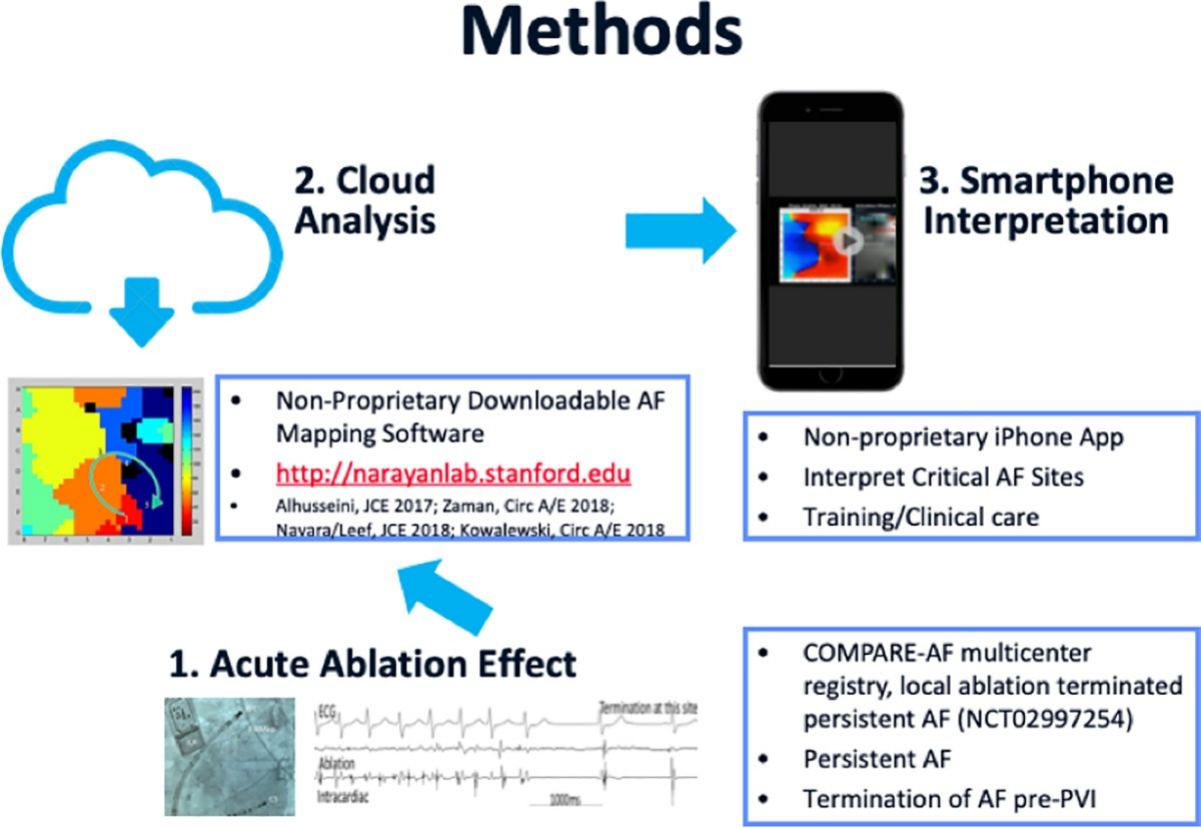
Proposed online training flow from App/Secure online server.

The main purpose of our study was to assess whether training on a complex clinical task–interpretation of panoramic AF maps–could be delivered through a mHealth approach. As we were able to demonstrate that this was feasible and effective, we moved on to migrate this concept to a smartphone App, so that we could widen its reach and potential impact. We were able to conduct internal testing on both an alpha and beta version of a prototype (4 individuals), with encouraging results. Despite being in its early stages, this smartphone App is among the first with a cloud computing back end to improve treatment of arrhythmia by improving diagnostic skills from novel mapping tools. Fig 7 shows potential scalability of this online-mobile paradigm.

For the prototype currently under development, our goal was to use a freely available online method (http://narayanlab.stanford.edu) and a commercial one–as tested in this trial–with others already planned for the near future.

Currently, there are few smartphone apps in the market for EP purposes, especially those targeted towards physicians rather than general information for patients.

## Limitations

In this proof-of-concept randomized trial, enrollment was limited to a small number of participants. This was mitigated, in part, by analyzing many maps, and the fact that are our results are intuitively correct. However, these results require validation in a larger trial, with different datasets and potentially more diverse mapping systems.

There is controversy on the contribution of AF sources as targets for ablation, yet results have been promising in several meta-analyses, and perhaps as a result of positive clinical studies the number of systems providing such maps is proliferating. Indeed, this motivated our study. Nevertheless, our approach of using digital online training methods in a graded fashion has general applicability and can have a bearing on several complex visual tasks in electrophysiology. This proof-of-training paradigm was a central goal of this study.

The sample was underpowered to assess participants’ performance and learning curve in identifying different types of drivers/sources.

## Conclusion

In this randomized-controlled trial we showed that automated structured training via an online webinar improved diagnostic skills on a challenging visual task, when compared to no training–interpreting complex panoramic AF maps to identify potential drivers or sources where ablation had been shown to terminate AF. Training improved accuracy for two AF mapping modalities (both a freely available and a commercial one).

Migration of this novel training paradigm to a custom Smartphone App accessing maps from the cloud, on a secure server, has been shown feasible. This novel online-mobile training paradigm represents a potentially powerful, scalable tool for complex training tasks in EP.

To the best of our knowledge, this was the first study to use online training for visual/movie EP tasks and demonstrate that this is effective for such data. By improving performance on challenging visual tasks, this may yield patient benefits.

## Supporting information

S1 Video This video exemplifies how the smartphone app for *AF map training* works and how users can navigate and access its multiple functionalities, such as watching tutorial movies, access PubMed indexed articles, take training/evaluation sessions and assess their own performance. (MP4)

S1 Table Comparison of test score according to previous gaming exposure. Readings were only accounted for the testing sets–training performance is not included.
(DOCX)

S1 Database File containing all the relevant data from this project, namely participants’ characteristics and performance, as well as main findings from our research. (XLSX)
