# Supplementary material for: Online webinar training to analyse complex atrial fibrillation maps: A randomized trial
Source: PLoS One. 2019 Jul 3;14(7):e0217988. doi: 10.1371/journal.pone.0217988 (PMC6609132; doi:10.1371/journal.pone.0217988)
Supplement: S1 Table — Readings were only accounted for the testing sets–training performance is not included. (DOCX) [file pone.0217988.s002.docx]

**Supplementary table** - comparison of test score according to previous gaming exposure. Readings were only accounted for the testing sets – training performance is not included.

| ***Group*** | ***Gamer*** | **Global results** | | | **Method A** | | | **Method B** | | |
| --- | --- | --- | --- | --- | --- | --- | --- | --- | --- | --- |
|  |  | Initial test score (%) | Final test score (%) | Delta (%) | Initial test score (%) | Final test score (%) | Delta (%) | Initial test score (%) | Final test score (%) | Delta (%) |
| **Control** | No  (n=4, 128 readings) | 31±6 | 30±9 | 0±3 | 36±13 | 37±15 | 1±3 | 25±8 | 26±7 | 1±5 |
|  | Yes  (n=2, 64 readings) | 43±4 | 49±4 | 5±0 | 47±4 | 61±0 | 14±4 | 39±4 | 40±4 | 0±0 |
| **p-value^[[1]](#footnote-1)^1** | | 0.076 | 0.058 | **0.041** | 0.331 | 0.110 | **0.010** | 0.078 | 0.084 | 0.745 |
| **Intervention** | No  (n=3, 96 readings) | 30±7 | 43±3 | 14±5 | 33±6 | 46±3 | 13±3 | 26±9 | 39±3 | 12±1 |
|  | Yes  (n=3, 96 readings) | 44±7 | 57±5 | 13±3 | 50±6 | 63±9 | 13±6 | 39±11 | 47±5 | 9±6 |
| **p-value^[[2]](#footnote-2)^1** | | 0.065 | **0.012** | 0.795 | 0.210 | 0.067 | 0.579 | **0.021** | **0.033** | 1.000 |
| **Cohort** | No  (n=7, 224 readings) | 31±6 | 36±10 | 5±8 | 35±10 | 41±12 | 6±7 | 26±8 | 32±9 | 6±8 |
|  | Yes  (n=5, 160 readings) | 44±5 | 54±6 | 10±5 | 49±5 | 62±6 | 13±5 | 39±8 | 44±6 | 5±6 |
| **p-value^[[3]](#footnote-3)^2** | | **0.003** | **0.003** | 0.282 | **0.018** | **0.013** | 0.863 | **0.010** | **0.003** | 0.065 |

1. 1 For comparison between *gamer* vs *non-gamer* performance within each group [↑](#footnote-ref-1)
2. [↑](#footnote-ref-2)
3. 2 For comparison between *gamer* vs *non-gamer* performance for the entire cohort, regardless of training (randomization effect) [↑](#footnote-ref-3)
